# Dental Research in the Digital Age: The Registry‐Based Clinical Trial

**DOI:** 10.1111/clr.70061

**Published:** 2025-10-24

**Authors:** Tim Joda, Eugenia Settecase, Lisa Heitz‐Mayfield, Jan Derks, Ronald E. Jung, Nicola U. Zitzmann

**Affiliations:** ^1^ Clinic of Reconstructive Dentistry, Center for Dental Medicine University of Zurich Zurich Switzerland; ^2^ Department of Reconstructive Dentistry University Center for Dental Medicine Basel, University of Basel Basel Switzerland; ^3^ International Research Collaborative, Oral Health and Equity, School of Human Anatomy and Biology University of Western Australia Crawley Western Australia Australia; ^4^ School of Dentistry, Faculty of Medicine and Health University of Sydney Sydney New South Wales Australia; ^5^ Department of Periodontology, Institute of Odontology, Sahlgrenska, Academy University of Gothenburg Gothenburg Sweden

**Keywords:** artificial intelligence, big data, dentistry, digitalization, evidence‐based medicine, public health, randomized controlled trial

## Abstract

With the global increase in the volume of digital health data recorded and accessible through national and institutional databases, such as clinical registries and evidence‐based registries, new strategic approaches are now feasible in medical research. These approaches include the registry‐based clinical trial (RBCT) design, where large‐scale datasets—which grow exponentially over time (referred to as big data)—can be used to identify eligible study participants from a medical registry containing trial‐specific inclusion criteria. The RBCT approach may also be used to establish historical control groups for prospective interventional studies that enable rapid recruitment with a lower study budget, while providing high statistical power. Hence, obstacles frequently encountered when conducting randomized controlled trials, such as difficulties in recruiting a sufficient sample size in a reasonable time period, may be overcome for specific research questions. This innovative study design of an RBCT aims to combine the external validity of medical registries with the internal validity of the traditional study designs, and has the potential to influence clinical decision making and healthcare policy. The aim of this perspective article is to describe this new methodological approach and to critically analyze the future possibilities and challenges of RBCTs in dental and implant research.

## 1 Introduction

Clinical trials are considered the pinnacle in medical research, with randomized controlled trials (RCTs) at the top of the evidence pyramid. RCTs generate rigorous evidence to support decision‐making and represent the gold standard in clinical research. By definition, the rationale of an RCT is to test the efficacy of one or more randomly assigned interventions or treatment modalities against a control procedure (as standard‐of‐care) on a defined outcome (Bergqvist et al. 2007). Researchers have to define the population, the interventions to be compared, and the outcomes of interest. The number of participants required to reliably determine whether the new intervention has an effect is calculated (sample size power calculation). The participants are then recruited and allocated to either the test or control intervention. To minimize bias, grouping is performed randomly, and participants as well as assessors are preferably unaware (blinded) of group allocation (Hariton and Locascio 2018).

In an ideal setting, the theory of an RCT is excellent due to its well‐controlled conditions (efficacy data), but problems often arise in practice due to the organizational complexity. The main drawbacks of RCTs can be narrow eligibility criteria, which often limit recruitment to a highly selected patient population. This can be addressed by multicenter designs, which require elaborate calibration of researchers from different clinical settings. Strict eligibility criteria may also lead to extended recruitment periods and long study durations, which may introduce further bias due to changes in framework conditions. In addition, RCTs often require an extensive level of human resources and incur high costs. Finally, RCTs can suffer from a high risk of failure and a restricted external validity, limiting conclusions on effectiveness in clinical practice settings—if ideal conditions cannot be realized (Lauer and D'Agostino 2013).

Today, many research groups plan and conduct RCTs, adhering to tradition and aiming for publication in prestigious scientific journals. Many of these RCTs, however, are underpowered, probably owing to the incentives to publish faster and reduce costs while receiving recognition for an RCT design (Pammi et al. 2025). Underpowered RCTs lack the necessary sample size to accurately detect meaningful effects, which can reduce the validity of the results obtained; such trials can have limited scientific impact and informative value (Hariton and Locascio 2018). While these RCTs with relatively small sample sizes may lack sufficient power, their results can still be valuable when combined in meta‐analyses and systematic reviews.

**FIGURE 1:**
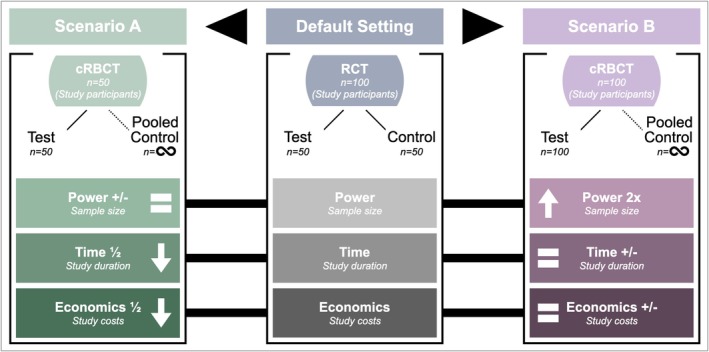
Overview of the key elements for the organization of a registry‐based clinical trial (RBCT).

With the global increase in the volume of digital health data, new strategic approaches are feasible in medical research. Registry‐based clinical trials (RBCTs), utilizing big data, are an innovative and forward‐thinking methodological approach to overcome the inherent challenges of conducting RCTs (Joda et al. 2018; Mathes et al. 2018). An essential prerequisite for conducting RBCTs is a solid source of structured (electronic) data, for example, from previous RCTs that have been entered into a clinical registry in a standardized way. Principles of classic RCTs can then be linked with the information content of the registry in order to create new prospective clinical study designs (Karanatsios et al. 2020; Shiely et al. 2024; Troxel and Hade 2024) (Figure 1).

The aim of this perspective article is to present a new methodological approach for designing and conducting clinical studies that ideally combines the external validity of clinical registries with the internal validity of classic trial designs, and second, to critically analyze the future possibilities and limitations of these RBCTs in dental research.

## 2 Data Source

Today, huge volumes of digital health data are collected from various sources, including routine dental processes via patient electronic health records (EHR), clinical research data based on electronic case report forms (eCRF), and also from extended sources, such as personal health apps, wearable devices, social media, and online behavior including digital commerce, namely the internet of things (IoT) (Joda et al. 2019).

### 2.1 Clinical Registry

The basic prerequisite for conducting RBCTs is access to structured patient data—ideally in electronic form. The classic data source is a clinical registry. Such a registry is characterized as an organized system that follows the principles of observational study methods to collect uniform ehealth data of a particular population defined by a disease, a condition, a specific treatment modality, or a combination of these (Liu et al. 2017). In general, a clinical registry serves one or more predetermined scientific, administrative, societal, or policy purposes. Clinical registries can therefore collect data for a particular area of interest, and depending on the depth of clinical details, they can address a variety of research questions. Longitudinal follow‐up within a clinical registry, as well as potential linkage to other sources of routinely collected data, such as EHRs or matching across different registries (e.g., in the field of systemic health), could even enable a deeper analysis of real‐world patients. However, registries are limited to their specific purpose, that is, they cannot cover all possible variables. The scientific output of RBCTs can mainly report on associations and only hypothesize about causality (McCord et al. 2018).

The structure of a clinical registry and its underlying purpose are decisive for its potential use in clinical research. Such a registry could be operated by a multi‐centric structure or by a single (academic) institution, for example, the Implant Registry at the Clinic for Reconstructive Dentistry at the Center for Dental Medicine at the University of Zurich. In the case of an institutional registry, the re‐identification of the patients involved could be guaranteed, as the data security factor is provided using a closed system. In a broader context, national health databases that exist in Scandinavia are another example of large‐scale clinical registries with a completely different structure and intention (SKaPa 2023). To date, there are no ideal structures for clinical registries per se. Each entry form has its own advantages and disadvantages.

The following section provides various examples of existing medical registries. Due to the health economic structure in Scandinavia, registry‐based studies are increasingly being conducted here in particular. In Sweden, Finland, and Norway, national registries covering the entire population are already established. As reimbursement for dental care, including implant‐supported prosthetic therapy, is administered by national bodies, for example, Social Insurance Agencies, treatment codes can be used to track large and real‐world patient populations. In another example, the FinRegistry project aimed to utilize nationwide health data to explore comprehensive risk trajectories of disease outcomes for the period 2021–2025, and used this information to develop statistical and machine learning models to predict disease occurrence. This project used the complex relationships between health outcomes, medication, socio‐demographic information, and familial risk to generate new epidemiological and biological hypotheses that can then be directly followed with more targeted studies. The results of this study should support future research into the development of personalized healthcare based on disease‐specific predictive models (https://thl.fi/en/research‐and‐development/research‐and‐projects/finregistry). The Norwegian Patient Registry collects information on all patients referred for specialized healthcare provided at a hospital, outpatient clinic, or by contract specialists (https://helsedata.no/en/forvaltere/norwegian‐institute‐of‐public‐health/norwegian‐patient‐registry‐npr/). The Swedish Quality Registry for caries and periodontal disease (SKaPa 2023) retrieves data from electronic patient files daily, facilitating the assessment of dental status in relation to general medical condition (e.g., diabetes) in more than 86,000 individuals, or evaluating the risk of tooth loss in case of furcation involvement in more than 380,000 subjects with almost 2.4 Mio molars (Trullenque‐Eriksson et al. 2023, 2024). A direct example in the field of implant dentistry is a register‐based cohort study analyzing the association between type 1 and type 2 diabetes and peri‐implantitis in 19,000 patients using multiple nationwide Swedish registers (Trullenque‐Eriksson et al. 2025).

It should be noted that data from nationwide registries represent treatment outcomes attained in daily practice in the general population, that is, effectiveness. Success rates may thereby be lower, and complication rates higher when compared with evaluations of efficacy in highly selected populations recruited to clinical trials, in which participants are typically treated by specialized clinicians in university or other expert settings (Zitzmann et al. 2011). The major advantage of large‐scale nationwide registries is the potential of linking dental and systemic health data. For example, previously unsuspected risk factors could be identified, bi‐directionally. On the other hand, there is a risk that data are not recorded in a standardized and structured manner and that traceability may be difficult due to anonymous recording.

### 2.2 Evidence‐Based Registry

While clinical registries are organized systems based on observational study methods, the so‐called evidence‐based registries are created from already published scientific findings of clinical trials. Clinical studies are conducted on a wide variety of topics in all areas of dentistry, and most of the results are accessible via multiple channels, such as medical search engines, scientific journals' databases, and social media. An established and proven instrument for synthesizing findings from multiple studies to facilitate clinical decision making on a focused clinical question is a systematic review including meta‐analysis. In this context, an evidence‐based registry can be seen as similar to an overarching systematic review providing historical control data. The results obtained from those control groups from multiple RCTs can be combined as pooled controls and utilized as a reference population for an evidence‐based registry study, creating a clinical trial with one prospective interventional test group and utilizing controls from an evidence‐based registry. At present, it would be a major challenge to synthesize such a registry from the multitude of publications as a structured data source. Continuous technological progress, however, will enable the automated analysis of scientific publications, not only for specific keywords but also for content directly related to particular research questions, using natural language processing (NLP) powered by artificial intelligence (AI) and machine learning (ML) (Joda et al. 2021; Locke et al. 2021; Eggmann et al. 2023).

### 2.3 Data Quality

The quality of the evidence‐based registry data pool is the critical factor for RBCTs. Setting up and maintaining ehealth registries can be time‐consuming, and the most common problems arise from underestimating methodological challenges, a lack of willingness to participate (by both subjects and researchers), insufficient resources and funding, as well as ethical issues related to the collection and storage of sensitive patient information. However, once a medical registry has been successfully established, the data can be updated and reused on repeated occasions as a valuable source for future RBCTs (Li et al. 2016).

**FIGURE 2:**
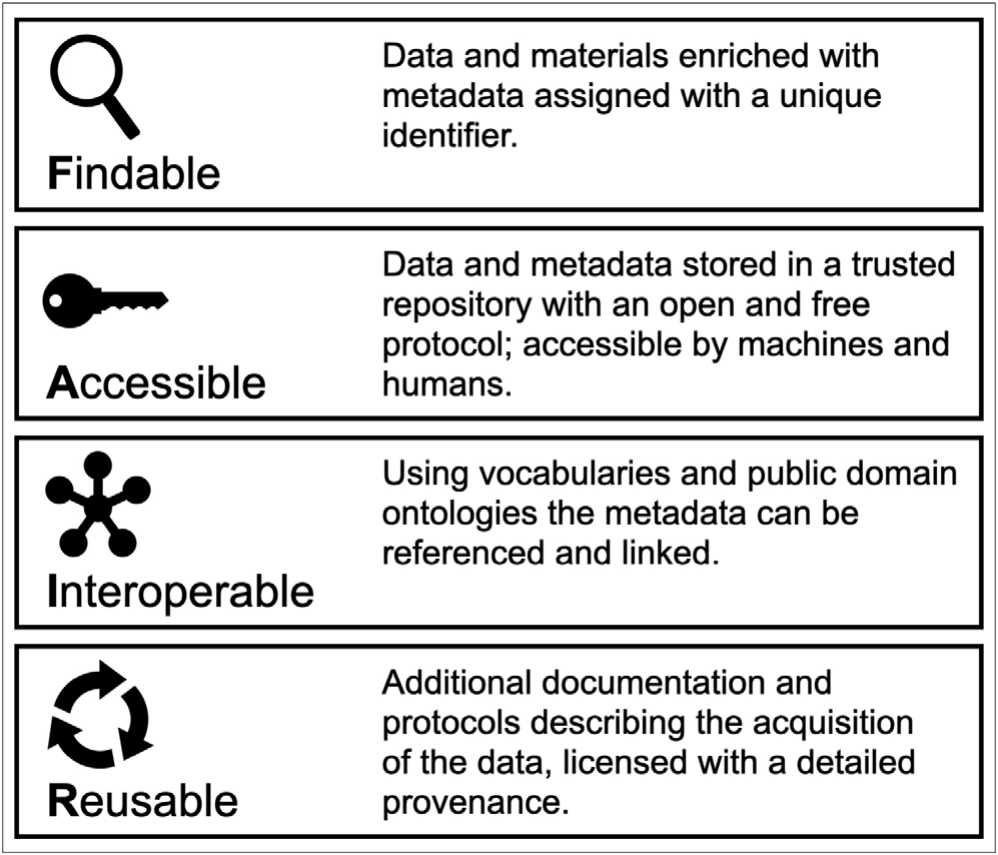
Definition of FAIR data principles (modified from: https://www.Go‐fair.org).

Clinical and evidence‐based registries should follow the internationally accepted FAIR principles. FAIR data are data that meet principles of Findability, Accessibility, Interoperability, and Reusability (https://www.go‐fair.org; Figure 2). Adhering to these principles will facilitate effective and efficient data collection, management, and sharing, while fostering collaboration and innovation in dental research. This compliance will make it possible to achieve real benefits for science and for society in a broader context.

## 3 Registry‐Based Clinical Trial (RBCT)

RBCTs can be defined as clinical trials in which eligible study participants are identified and recruited from a clinical or an evidence‐based registry containing specific eHealth data. RBCTs combine traditional clinical trial methodology with the advantages of registry systems to gain new clinical insights (Foroughi et al. 2018). This setup allows for faster recruitment by screening patients retrospectively who can then be included in prospective trials. If general informed consent is available, data from earlier RCTs with follow‐up information can also be reused. This avoids repeating interventions that carry known risks. For example, to assess a new, narrow‐diameter implant for replacing a lower incisor, only one interventional test group may be necessary. Its results can then be compared with standard implant data already available in the registry. From an ethical point of view, this approach is reasonable, as it may prevent exposing patients to outdated or inferior treatments, such as older diameter‐reduced implants made of a weaker material.

Depending on the structure and depth of clinical information captured and collected in the registry, RBCTs can support a variety of research questions. RBCTs are particularly suited for testing hypotheses involving already available and established clinical protocols, interventions, diagnostics, or pharmaceutical products in a real‐world environment. Here, RBCTs are helpful when there is uncertainty about optimal treatment combinations, sequences, or durations, or when multiple standard‐of‐care options exist. RBCTs are cost‐effective to pursue clinical research questions while maintaining trial rigor (Mathes et al. 2018).

### 3.1 Randomized Registry‐Based Clinical Trial (rRBCT)

**FIGURE 3:**
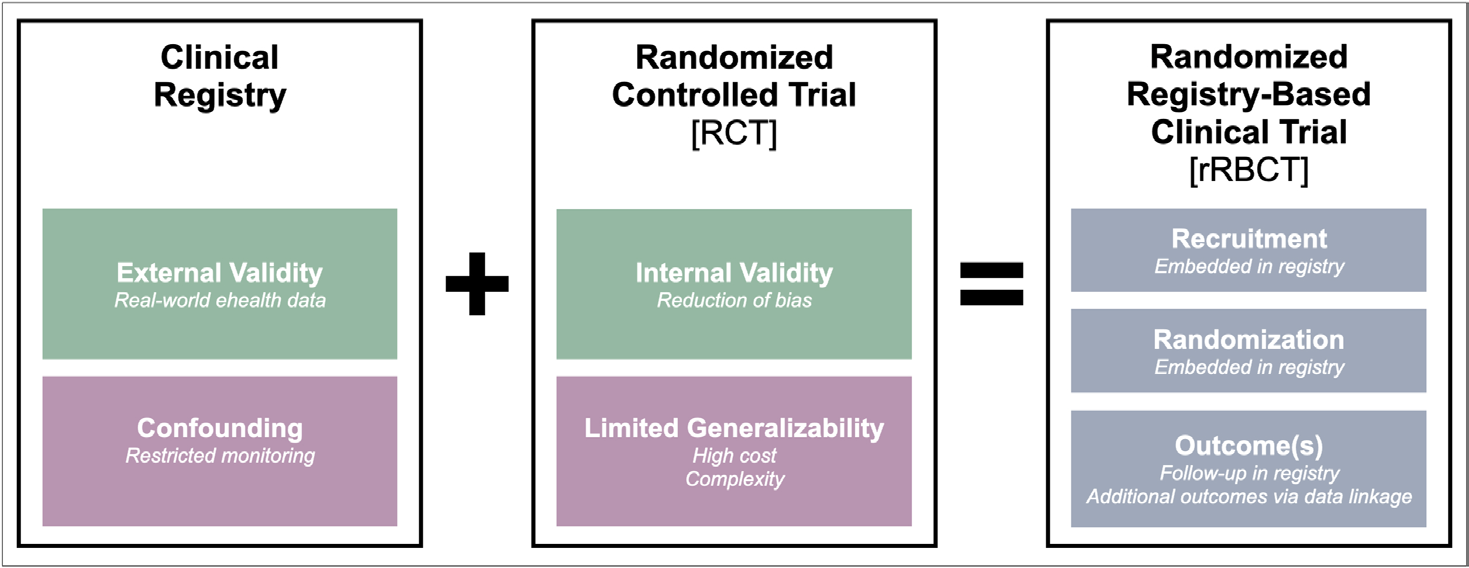
Synthesis of an rRBCT out of a clinical eHealth registry and a traditional RCT.

In the past, the development of a variation to the traditional RCT has been reported, namely a randomized registry‐based clinical trial (rRBCT) (Asberg et al. 2017; Ragnarsson et al. 2020), in which the classic structure of an RCT is embedded in a patient registry (James et al. 2015; Li et al. 2016). This innovative approach combines the high internal validity of RCTs with the high external validity (applicability in a clinical setting) of real‐world ehealth data obtained through registries (Troxel and Hade 2024) (Figure 3).

Optional add‐on trial modules can be included in the registry to enable (new) randomization and collection of trial‐specific data, such as patient‐reported outcomes. These data are collected as part of routine care, using the registry's infrastructure to capture baseline and outcome information. This design allows for broad inclusion criteria, facilitating access to a diverse patient population and enabling rapid recruitment. This allows rRBCTs to be conducted with broad eligibility criteria, enabling access to studies for a large group of participants who reflect real‐world patients. The costs of such trials are typically much lower than those of traditional RCTs (Shiely et al. 2024).

There are several ways in which registries are used in rRBCTs. Some serve a single purpose, such as screening of potential participants or identifying outcomes (Katapodi et al. 2013), while others support multiple steps of the trial process, including recruitment, data collection, or randomization of cases (Sundh et al. 2019). In this context, the rRBCTs are described as trials, where the registry serves as a platform for both participant recruitment and data collection including the acquisition of outcome and endpoint data (Li et al. 2016).

### 3.2 Cohort Registry‐Based Clinical Trial (cRBCT)

Not all RBCTs require randomization to a test or control group. In many areas of dental research, existing RCTs already include similarly structured control groups. These historical datasets can be pooled to create external comparators for new trials. This allows the design of cohort registry‐based clinical trials (cRBCTs), where only a new intervention group is tested, and outcomes are compared to matched data from historical controls (Schmidli et al. 2020). This matching can be supported by modern computer technology, such as AI and ML.

In cRBCTs, participants are retrospectively selected from the registry, and prospective intervention can proceed without creating a new control group. Instead, the historical control group is used for comparison. This saves time, reduces costs, and produces quicker results that can benefit clinical care. However, precise matching protocols are essential to ensure valid comparisons and reduce bias (Pocock 1976).

Although RCTs remain the gold standard for evaluating the safety and efficacy of new treatments, ethical or practical issues sometimes make it difficult to establish adequate control groups. When previous evidence suggests a new therapy is superior, it may not be ethically justifiable to expose patients to an older, potentially less effective standard treatment (Favaretto et al. 2020). Thus, cRBCTs are a useful alternative, allowing new therapies to be tested against well‐documented historical data. These data also provide a solid basis for power calculations and sample size estimations at the study's outset.

**FIGURE 4:**
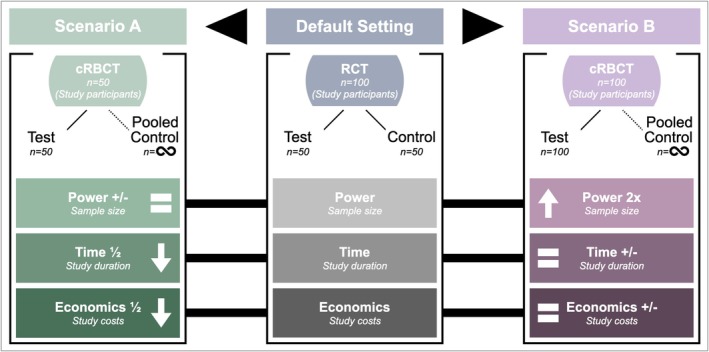
Scenarios for the design of a cohort registry‐based clinical trial (cRBCT) considering the variables: (1) power (sample size); (2) time (study duration); and (3) economics (study costs).

Figure 4 illustrates the potential of cRBCTs. A classic RCT design with three variables is shown: (1) power (sample size), (2) time (study duration), and (3) economics (study costs). When conducting a cRBCT with the same sample in the test group (Scenario A), the power logically remains the same compared to the RCT, but both the study duration and study costs are halved. On the other hand, the power can be amplified by doubling the sample size in the test group (Scenario B). Study duration and study costs remain unchanged.

The scenarios presented in Figure 4 are extreme examples of the impact of administrative (Scenario A) and statistical (Scenario B) variables associated with the design and planning of a cRBCT. The power is affected by the sample size, but it is also affected by additional variables, such as type I error and effect size. By splitting Scenario B into two cases, the peculiarities of sample size calculations and the influence of statistical variables are explained.

When calculating a required sample size for a clinical trial in general, researchers pre‐define these variables according to the specific research question and available information within a hypothesis. The formula for calculating the sample size depends on the statistical test used for the proposed hypothesis (Ranganathan et al. 2024). Hence, for this example, a two‐arm clinical trial is considered (test versus control). The null and alternative hypotheses are compared using a two‐sample *t*‐test. The null and alternative hypotheses read as follows:

$$(H_{0})\;\mu_{\text{control}}=\mu_{\text{test}}$$

$$(H_{1})\;\mu_{\text{control}}\neq \mu_{\text{test}}$$

**FIGURE 5:**
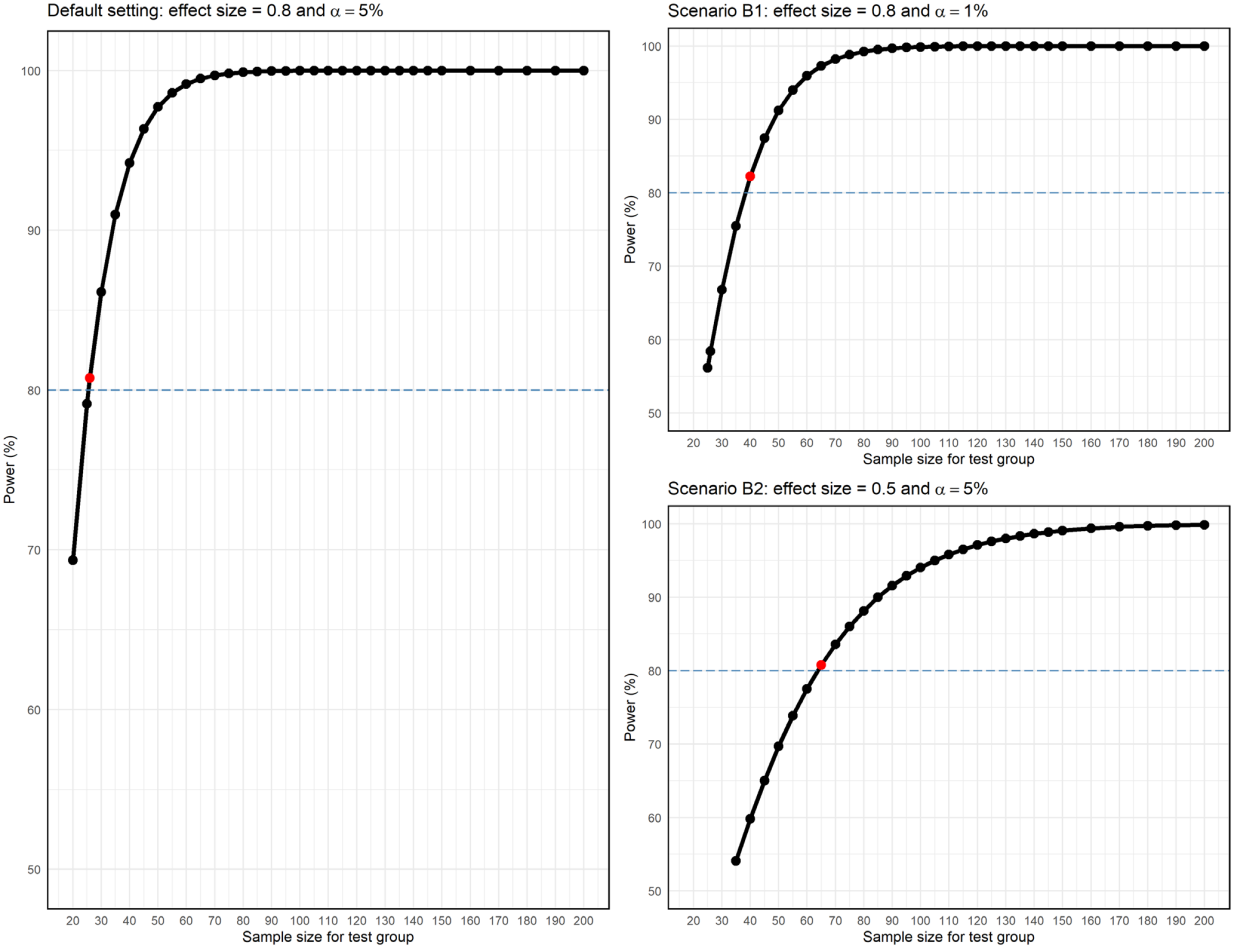
Repercussions of the changes in statistical variables on the required sample size for Scenario B, as previously illustrated in Figure 4. In a hypothetical example for a sample size calculation of the test group (Default Setting) with a desired power of 80%, an *α* of 0.05 and a large effect size of 0.8, the necessary sample size is *n*_test_ = 26. In the two Scenarios, B1 and B2, the power remains unchanged from the default setting at 80%, while the variables *α* and the effect size varies accordingly. For Scenario B1, *α* is reduced from 5% to 1% with a fixed effect size of 0.8, the required sample size is *n*_test_ = 40. For Scenario B2, the effect size changes from large (0.8) to small (0.5), with a fixed *α* of 5%, the required sample size is then *n*_test_ = 65. For all scenarios, it can be seen that the effect of an increase in power has a direct effect on the increase in sample size following a non‐linear curve.

The type I error, also known as α, is defined as the probability of incorrectly rejecting $H_{0}$ when it is true, while the statistical power is defined as 1−β, where β is the type II error, the probability of incorrectly rejecting $H_{1}$ when it is true. The effect size determines the difference that the statistical test can detect between the means of the study groups (test versus control; Figure 5).

### 3.3 RBCT Checklist

Setting up an RBCT follows similar principles to those of a classic clinical trial, but additional registry‐specific aspects have to be considered. Table 1 summarizes the ten most important factors serving as a checklist and basic guideline for the initial planning and implementation of an RBCT (https://vcccalliance.org.au). RBCTs vary depending on the scientific question and the linked data source of the specific registry. Therefore, this checklist can only provide a rough overview and should not be considered conclusive.

**TABLE 1:** Checklist and guideline from planning to implementation and publication of an RBCT (modified from: https://vcccalliance.org.au).

Phases	Clinical registry	r/cRBCT	
		Setting up a trial in an existing registry	Developing a new registry in conjunction with a trial
[1] Planning	Definition of the particular cohort and outcomes	Is the existing registry designed to capture a patient population relevant for the planned trial?	Can a new registry be designed and established in a timely and cost‐effective manner?
	>> *Registry concept*	>> *Focused study question*	>> *Focused study question*
[2] Protocol	The aims and objectives of the registry must be determined in a separate protocol	The trial needs a standalone protocol (even when it is running through an existing registry)	Trial plus registry protocol development (the trial needs a standalone protocol)
	>> *Registry protocol*	>> *Study protocol*	>> *Study protocol*
[3] Ethics	Approval from the responsible ethics committee (for all possible sites involved)	The study protocol must be approved by the responsible ethics committee	Both protocols (study plus registry) must be approved by the responsible ethics committee
[4] Database	Software and data points to be recorded must be defined	If additional data will be assessed in the trial, mechanisms for capturing these data must be defined	The database must fulfil the requirements of the standards of the trial and the registry
[5] Site registration	Decision on which sites and institutions the registry is implemented	Decision on which sites and institutions the trial is implemented (if multiple sites are involved in the registry)	All participating sites take part in the trial and the registry
[6] Governance	Each site involved in the registry must formalize participation through a research governance office (RGO)	Each site involved in the trial must formalize participation through a research governance office (RGO)	
[7] Patient enrollment	Systematic process for identifying patients for inclusion in the registry	All patients must be identified and consented for the trial	
[8] Data capture	Data recorded must be accessed and stored by delegated project officers	Study coordinators must be appointed at each site with a clear demarcation of trial responsibilities from the registry	Study coordinators must be appointed at each site, assisting in trial and/or registry implementation
[9] Data use	Secure way to store and share de‐identified data centrally	Study‐related data are extracted from the registry for all patients for analysis	
	>> *Centralized data storage*	>> *Data analysis*	
[10] Reporting		Making results accessible by publishing them in scientific journals and/or presenting at congresses, etc.	

### 3.4 Limitations of RBCTs

There are several challenges in the design and implementation of RBCTs that need to be addressed, including data quality, regulatory and ethical issues, adjudication of study outcomes, choice of methodology and study design, and operational challenges emanating from the type of clinical registry and routinely collected ehealth data used (McCord et al. 2018).

In terms of the methodological approach within RBCTs, selection bias related to controls certainly needs to be considered. It should also be considered that the researchers involved need to be trained and calibrated on how to include the required information in the specific registry. Researchers should also be aware of the potential for bias arising from the appearance of false patterns due to prior knowledge of the data (Tierney et al. 2021). In addition, temporal effects and heterogeneity within and across groups must also be evaluated. As trial participants used as controls originate from historical trials, inherent differences when compared to the test groups may exist. These may be due to changes in terms of diagnostics, standard of care, and technical tools, external factors, and demographics. To avoid critical discrepancies between groups, the inclusion criteria must be strictly defined and adhered to. The selection of historical studies that are to serve as potential controls should also consider the aim of the study and corresponding outcomes, the target population, and the clinical protocols. Equally important is the access to (and quality of) the available data from such studies. The selection process for control data, therefore, plays a key role in the quality and overall validity of any RBCT (Gray et al. 2020).

## 4 Open Research Data Platforms

From an economic point of view, it makes little sense to set up, establish, and manage multiple isolated registries at different centers. A meaningful alliance with collaborative networking offers many new opportunities in clinical dental research. Synergies can also arise in those new solutions. Treatment methods or risk factors for a health condition can be identified at an earlier stage by holistically collecting and analyzing ehealth data. Health information systems should be strengthened to collect and disseminate mobile health data enabling analytics for clinical as well as strategic decisions (Joda et al. 2018, 2019).

The goal must therefore be to exploit possible synergies and initiate large‐scale registries as open research data platforms. If multi‐disciplinary clinical registries are established, dental and oral factors could be linked with systemic parameters. National health registers, as in Scandinavia, are predestined for this, as individuals in the system have an anonymized but consistent and unique identification number for both medical and dental treatment.

The first step is to ensure the visibility of existing registries and to create an overview and disseminate information on the general topic, to generate geographical coverage, potential participation opportunities, data access, reporting structures, and contact (https://www.nih.gov/health‐information/nih‐clinical‐research‐trials‐you/list‐registries). Such a database could serve to increase the networking of registry operators and transparency in the field, but also to provide guidance for the future, accessible to the public, when setting up new registries.

However, the theoretical framework is far simpler than the actual realization. There are already major legal differences at the national level about data collection, management, and security, not to mention international legislation (Califf and Sugarman 2015). Therefore, legal restrictions on the conduct of an RBCT may not be easy to overcome when several countries are involved. Here, it should be emphasized that it needs to be clarified how the quality and possible veracity of the information can be controlled, especially when economic or political interests are involved. In addition to restrictions for the transfer of sensitive patient data, language barriers and discrepancies in healthcare systems come into play. Factors to consider are differences in terms of reimbursement systems and health care policies, for example, use of classification and clinical guidelines, as well as the level of professional (dental) education.

In this context, blockchain technology could be a door‐opener with the power to enable open research data platforms for sharing structured health data. The concept is essentially based on decentralized data that is stored in linked databases. Blockchain technology, based on security, data protection, and legal regulations for health data, would enable cross‐organizational services or workflows in real‐time for users inside and outside of national health systems anywhere in the world (Corte‐Real et al. 2022). Future important indications for using blockchain technologies in dentistry concern not only dental implants, but also the identification and treatment of syndromes with medical implications for the patients. Data protection and the informed consent by patients to disclose their data are of paramount importance. When setting up a registry, it can be a challenge to cover all potential future uses of the collected data, and patients may need to be consulted again for specific usage of their data in a future study.

## 5 Conclusions

RBCTs have the potential to enhance clinical research and can play a major role in dentistry by influencing healthcare services, research, education, medtech and pharma industry, insurance business, social policy, governmental affairs, and legal regulations. Continuous advancement in AI technology, such as ML and NLP, will probably fast‐track data acquisition and analysis in the near future.

RBCTs can have great value as they are built on large real‐world datasets. They have the power to increase recruitment efficiency and provide information on follow‐up, while also enabling the overall study cost to be critically reduced when compared with RCTs.

RBCTs should be seen as a complement to classic RCTs and cannot replace them in clinical research, but rather use existing data from RCTs. However, they can be a valuable addition and provide new insights or more broadly anchored information compared to RCTs. A new hierarchical field in the classical evidence pyramid would have to be created for RBCTs. Depending on the specific trial design of an RBCT, that is, retrospective or prospective, randomized or cohort‐based with historical controls, the position in the evidence pyramid would be different. Research findings obtained from RBCTs could be considered equivalent to a classic RCT or a cohort study.

The establishment of clinical registries is also not equally possible or useful in all areas of dental research. Implant registers are a good example, which can be set up and maintained relatively easily. Once a clinical registry has been successfully established, it can be used over and over again as a basis for possible new RBCTs. The power of such a registry would be expected to increase exponentially as the amount of data increases and the quality of data improves.

In summary, RBCTs have the potential to enable:

- High‐quality clinical research with large‐scale sample sizes and accelerated recruitment;
- Immediate access to clustered retrospective data;
- Systematic filtering of eligible patients for prospective research applying trial‐specific inclusion criteria;
- Generation of scientific evidence with low bias and high levels of external validity involving real‐world health data;
- Epidemiological surveys for public health‐related statistics;
- Evaluation of the interplay between oral and systemic health; and
- Trend research and early identification of future research needs.

## Author Contributions

**Tim Joda:** conceptualization, writing – original draft, methodology, validation, writing – review and editing, project administration, supervision. **Eugenia Settecase:** writing – original draft, methodology, validation, writing – review and editing, formal analysis. **Lisa Heitz‐Mayfield:** validation, writing – review and editing. **Jan Derks:** validation, writing – review and editing. **Ronald E. Jung:** writing – review and editing, resources. **Nicola U. Zitzmann:** writing – original draft, validation, writing – review and editing, supervision.

## Ethics Statement

The authors have nothing to report.

## Consent

The authors have nothing to report.

## Conflicts of Interest

The authors declare no conflicts of interest.

## Data Availability

This is a *perspective article* describing a novel method in dental research. No (patient) data are reported in this article.
